# Antibacterial activity testing methods for hydrophobic patterned surfaces

**DOI:** 10.1038/s41598-021-85995-9

**Published:** 2021-03-23

**Authors:** Ana Perez-Gavilan, Joana Vieira de Castro, Ainara Arana, Santos Merino, Aritz Retolaza, Sofia A. Alves, Achille Francone, Nikolaos Kehagias, Clivia M. Sotomayor-Torres, Donato Cocina, Renato Mortera, Salvatore Crapanzano, Carlos Javier Pelegrín, María Carmen Garrigos, Alfonso Jiménez, Begoña Galindo, Mari Carmen Araque, Donna Dykeman, Nuno M. Neves, Jose Maria Marimón

**Affiliations:** 1 Biodonostia, Infectious Diseases Area, Respiratory Infection and Antimicrobial Resistance Group, Microbiology Department, Osakidetza Basque Health Service, Donostialdea Integrated Health Organisation, 20014 San Sebastian, Spain; 2 3B’s Research Group, I3Bs—Research Institute On Biomaterials, Headquarters of the European Institute of Excellence On Tissue Engineering and Regenerative Medicine, Biodegradables and Biomimetics of University of Minho, AvePark—Parque de Ciência e Tecnologia, Zona Industrial da Gandra, 4805-017 Barco, Guimarães Portugal; 3 ICVS/3B’s - PT Government Associate Laboratory, Braga/Guimarães, Portugal and The Discoveries Centre for Regenerative and Precision Medicine, Headquarters at University of Minho, Avepark, 4805-017 Barco, Guimarães Portugal; 4 Tekniker. Iñaki Goenaga 5, 20600 Eibar, Spain; 5 Departamento de Electricidad y Electrónica, Universidad Del País Vasco, UPV/EHU, 48940 Leioa, Spain; 6 Catalan Institute of Nanoscience and Nanotechnology (ICN2), CSIC and BIST, Campus UAB, 08193 Bellaterra, Barcelona Spain; 7 Institució Catalana de Recerca i Estudis Avancats (ICREA), 08010 Barcelona, Spain; 8 Propagroup S.P.a. - R&D Department, via Genova 5/b, 10098 Rivoli (Turin), Italy; 9 Department of Analytical Chemistry, Nutrition & Food Sciences, University of Alicante, 03690 San Vicente del Raspeig, Alicante Spain; 10 AIMPLAS Technological Institute of Polymers, 46980 Paterna, Valencia Spain; 11 Materials Business Unit, Collaborative R&D Department, Ansys Inc, Cambridge, CB17EG UK; 12 Servicio de Microbiologia, Hospital Universitario Donostia, Paseo Dr Beguiristain s/n, 20014 Donostia-San Sebastián, Spain

**Keywords:** Biological techniques, Biotechnology, Microbiology

## Abstract

One strategy to decrease the incidence of hospital-acquired infections is to avoid the survival of pathogens in the environment by the development of surfaces with antimicrobial activity. To study the antibacterial behaviour of active surfaces, different approaches have been developed of which ISO 22916 is the standard. To assess the performance of different testing methodologies to analyse the antibacterial activity of hydrophobic surface patterned plastics as part of a Horizon 2020 European research project. Four different testing methods were used to study the antibacterial activity of a patterned film, including the ISO 22916 standard, the immersion method, the touch-transfer inoculation method, and the swab inoculation method, this latter developed specifically for this project. The non-realistic test conditions of the ISO 22916 standard showed this method to be non-appropriate in the study of hydrophobic patterned surfaces. The immersion method also showed no differences between patterned films and smooth controls due to the lack of attachment of testing bacteria on both surfaces. The antibacterial activity of films could be demonstrated by the touch-transfer and the swab inoculation methods, that more precisely mimicked the way of high-touch surfaces contamination, and showed to be the best methodologies to test the antibacterial activity of patterned hydrophobic surfaces. A new ISO standard would be desirable as the reference method to study the antibacterial behaviour of patterned surfaces.

## Introduction

Hospital-acquired infections (HAI) can be defined as the infections acquired by a patient who was admitted to a hospital or other healthcare facility that were not present (or incubating) at the moment of admission. HAI represent a significant healthcare problem with an estimated total of 8.9 million cases occurring in the EU during 2016–2017 in acute care hospitals and long-term care facilities^1^. The prevalence of HAI has been estimated to vary between 4.4% in primary care hospitals to 7.1% in tertiary care hospitals, reaching 19.2% in intensive care units^1^. In the EU in 2015, around 25% of HAI were caused by antibiotic-resistant bacteria with an estimated annual mortality of 33,000 patients^2^.

HAI can be caused by pathogens coming from other patients, the staff or the hospital environment. The different high-touch surfaces such as door handles, tables, nurse-call buttons or bed rails can be contaminated by potential pathogens which are able to form biofilms and survive on the surfaces for a long time^3^. It has been demonstrated that some antibiotic-resistant bacteria, as methicillin-resistant *Staphylococcus aureus* (MRSA) and vancomycin-resistant *Enterococcus* (VRE), are able to survive for weeks on different surfaces^4^. Recent evidence confirmed that the previous occupation of rooms by patients infected or colonized with MRSA, VRE or other antibiotic-resistant pathogens increased the risk of new patients to be colonized with those pathogens^5^. Consequently, appropriate protocols to clean and disinfect hospital surfaces are crucial to prevent HAI of which manual cleaning with disinfectants is the most commonly used. However, chemical cleaners used to disinfect surfaces are not exempt from certain degree of toxicity, can be incorrectly applied and have to be used at their effective concentration. Moreover, once applied on surfaces, disinfectant activity disappears and the objects could contaminate again within minutes^6^.

Modified surfaces can lead to a decrease in the bacterial attachment and biofilm production, which has raised interest in the research on polymeric films with structured surfaces with intrinsic properties or on films with embedded antibacterial agents. There is evidence that different polymers with specific micro- and nano-topographies can inhibit the attachment, growth and spread of microorganisms^7–9^. Furthermore, different polymeric materials treated with silver, copper, polycations, triclosan, bacteriophages or light activated biotoxic radicals have been developed^10^.

To assess the activity of films with antibacterial behaviour, appropriate evaluation tests are needed. The ISO 22196 standard (Japanese test method JIS Z 2801) is used for the measurement of antibacterial activity on plastic surfaces^11^. However, some studies have described this test as inappropriate, since the temperature of incubation (35 ± 1 °C) and the relative humidity (higher than 90%) do not reflect real conditions^12^. Thus, alternative and more realistic methods for the in vitro study of antibacterial activity of plastic surfaces have been described^7,13^.

In this study, the main objective was to compare the performance of different described tests to measure the antibacterial activity of plastic surfaces. To achieve this goal, the antibacterial activity of a three-layer polypropylene (PP) polymer matrix with two different nano- and micro-structures was evaluated with previously described tests (the ISO 22916 standard^11^, the immersion method^7^, and the touch-transfer inoculation method^7^). Moreover, a new methodology based on the protocols described by Mann et al*.*^7^ was designed for this study, called the “swab inoculation method”.

## Methods

### Preparation of film specimens

The test film comprised a smooth three-layer film made of two 25 µm external PP layers (SABIC grade, PP-2 SABIC PP 520L), and a 50 µm central layer of an olefin based thermoplastic elastomer (ZELAS 7025) produced at Propagroup (Torino, Italy). The inner layer was ready to function as a reservoir for the progressive release of encapsulated essential oils or other compounds with antibacterial activity. In this work, only structured films without antibacterial compounds were tested to exclusively check the antibacterial activity of patterned surfaces.

**Figure 1:**
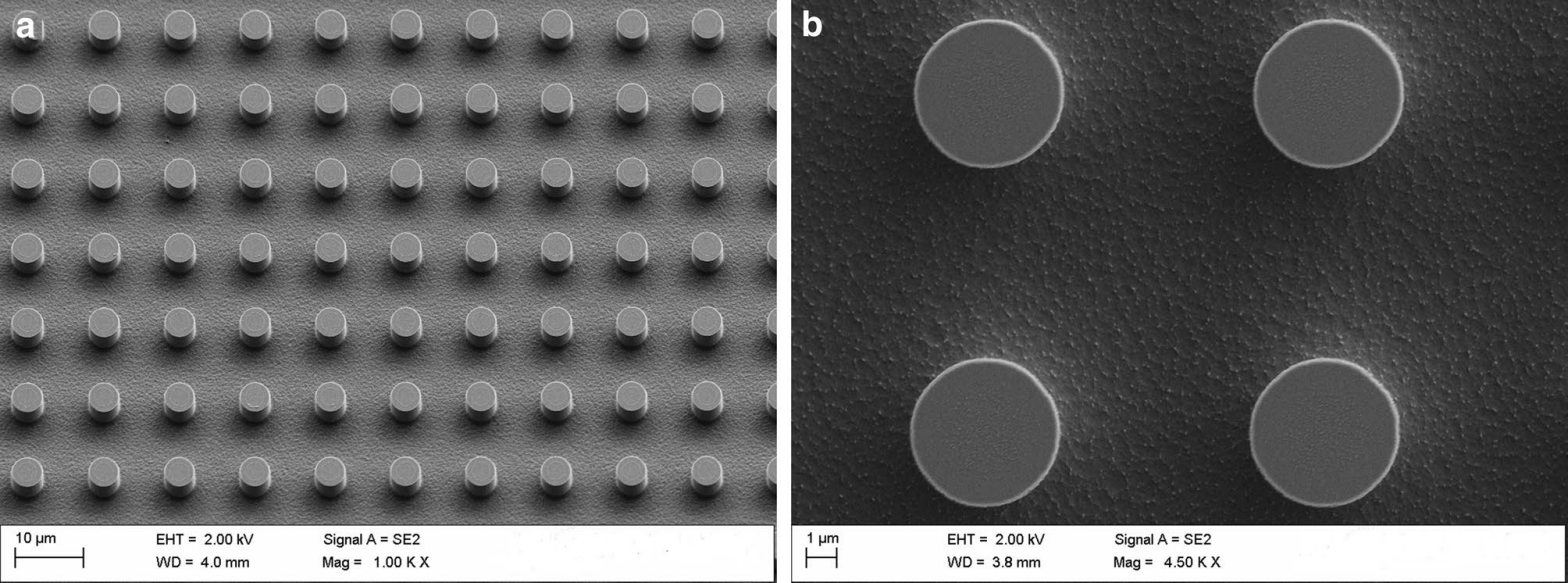
Topography of the structured films: 5 μm diameter micropillars fabricated on a PP substrate (left; 10 µm scale), where the roughness they show at the bottom corresponded to the presence of nanospikes (right; 1 µm scale).

A topography containing ordered micro- and nano-patterns was imprinted on the surface of the PP film by means of nanoimprint lithography. The topography consisted on 5 μm cylindrical micropillars with nanospikes covering the bottom, among micropillars (Fig. 1). A nickel stamp was used as a stamp, which was copied from an original silicon master^14^. Non-structured smooth films were used in the different tests as control.

### Bacterial strains and growth conditions

The bacteria used in this study were the Gram-negative *Escherichia coli* (ATCC 25922) and the Gram-positive *Staphylococcus aureus* (ATCC 25923). Bacteria from the frozen stock culture were transferred to Trypcase Soy Agar (TSA) supplemented with 5% sheep blood plates (TSA, bioMèrieux, France) and incubated at 37 °C for 18–24 h. Bacteria were transferred to 50 mL of sterile Tryptic Soy Broth (TSB) medium and grown at 37 °C, 80 rpm, 18–24 h. Prior to inoculation, strains were subcultured into fresh TSB at 1:50 and incubated for 2 h at 37 °C, 80 rpm. Bacteria were collected by centrifugation (2000 rpm, 10 min) and resuspended in phosphate buffered saline (PBS). The number of bacteria was spectrophotometrically adjusted to O.D._600 nm_ = 0.5 (equivalent to 1.5 × 10^8^ CFUs/mL) and confirmed by culturing 1/10 serial dilutions of the initial suspension.

**Figure 2:**
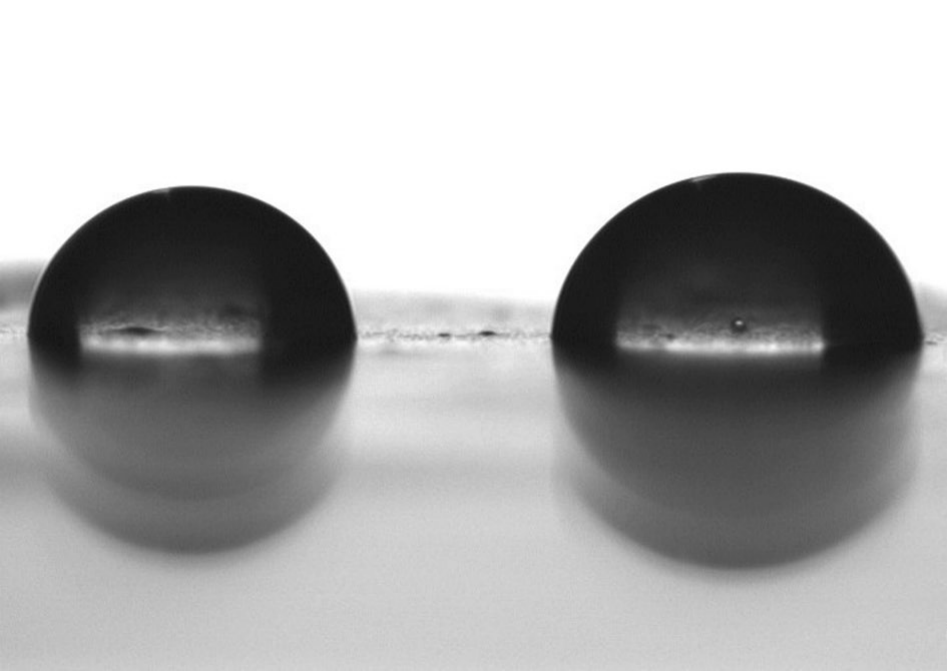
Hydrophobic behaviour of the PP film showing a high contact angle (97.8° ± 1.1°) of a drop of PBS (left) and a lower contact angle (86.4° ± 1.1°) of a drop of PBS with 5% glycerol (right) (p < 0.001) measured with a SURFTENS universal goniometer. The white rectangle inside the droplets is the reflections of the diffuse light illuminating the drop to obtain the image with a sharper border.

The patterned films evaluated in this study were highly hydrophobic so it was difficult to homogeneously spread the aqueous inoculum over the surface (Fig. 2 left). In order to resolve this problem, the bacterial inocula were applied using glycerol to reduce the surface tension of the suspension (Fig. 2 right). Three different final concentrations of glycerol were tested (5%, 10% and 40%). For all experiments it was decided to use the lowest concentration of glycerol (5%) as the distribution of the inoculum was as homogenous as with higher concentrations of glycerol (40%) and more homogenous than with PBS alone and the bacterial recovery was similar at all glycerol concentrations tested (data not shown).

### Antibacterial activity tests

**Figure 3:**
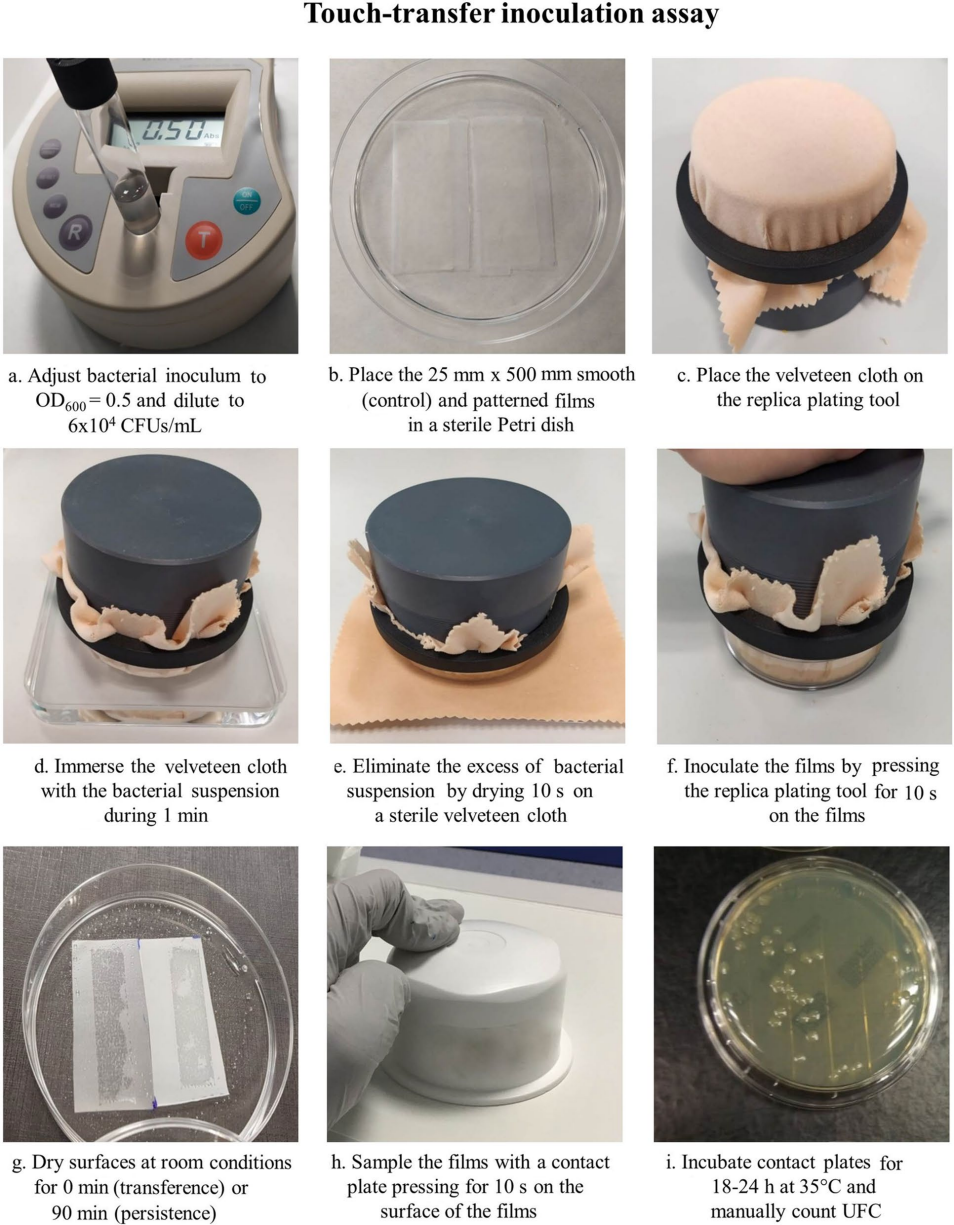
Schematic representation of the touch-transfer assay.
- **Swab inoculation assay**
  The Swab inoculation assay or Swab assay was designed for this study based in the Touch-transfer inoculation assay in order to obtain more reproducible results. This assay also assessed the attachment and survival of the bacteria on the surface of the film. Serial dilutions of the bacteria inoculum were made in PBS supplemented with glycerol 5% to reach a bacterial concentration of 6 × 10^4^ CFUs/mL (Fig. 4a). Fifty microliters of the bacterial suspension were pipetted on the control and patterned films (25 mm × 50 mm) (Fig. 4b) and spread homogenously by rolling a cotton swab over their surface (Fig. 4c). Films were allowed to dry under ambient conditions (± 24 °C and humidity of ± 35%) for 0 min (transference) or 90 min (persistence) (Fig. 4d) before being sampled (Fig. 4e) and colonies counted (Fig. 4f).

- **ISO 22196 standard method**
  The ISO 22196 was performed with slight modifications^11^. Pieces of 50 mm × 50 mm of the film and of non-structured smooth control films were placed in sterile 90 mm Petri dishes. Serial dilutions of the bacteria inoculum were made to reach a final bacterial concentration of 6 × 10^5^ CFUs/mL of which 400 µL were pipetted onto the test surfaces. Bacterial inoculum was then covered with 40 mm × 40 mm piece of STOMACHER bag in order to homogeneously spread the inoculum over the surface. The specimens were incubated for 24 h at 35 °C and a relative humidity > 90%. To recover the bacteria from the surfaces of the plastic samples, 10 mL of Soybean Casein Lecithin Polysorbate (SCDLP) broth was added to the Petri dish. The SCDLP was collected and released at least four times to ensure that the specimens were completely washed. Then, tenfold serial dilutions of the SCDLP were made in PBS and 50 µL of 10^–4^, 10^–5^, 10^–6^ and 10^–7^ dilutions were cultured on TSA with 5% sheep blood plates. Plates were incubated at 35 °C for 24 h. The number of colonies per plate was recorded and used to determine the number of viable bacteria per cm^2^ in accordance with the equation:
  $$N = \left( {100 \times C \times D \times V} \right)/A$$
  where C is the average of CFU count, D is the dilution factor, V is the volume in mL of SCDLP and A is the surface (cm^2^) of the film.
- **Immersion inoculation assay**
  Immersion inoculation assay^7^ evaluated the attachment and survival of the bacteria directly from the surface of the film. Serial dilutions of the bacteria inoculum were made in PBS to reach a bacterial concentration of 6 × 10^4^ CFUs/mL. Film samples (50 mm × 50 mm) were placed in a 90 mm sterile Petri dish and were completely covered with 20 mL of bacterial inoculum for 1 h at room temperature without shaking. The films were then washed 3 times with 20 mL of PBS for 10 s while rotating at 80 rpm and allowed to dry under ambient conditions for 1 h before being sampled.
- **Touch-transfer inoculation assay**
  The Touch-transfer inoculation assay was described by Mann et al*.*^7^ and assessed the attachment (transference) and survival of the bacteria (persistence) directly on the surface of the film. In this study, the protocol described by Mann et al*.*^7^ was used with minor changes. Briefly, serial dilutions of the bacteria inoculum were made in PBS to reach a bacterial concentration of 6 × 10^4^ CFUs/mL (Fig. 3a). This inoculum was experimentally established on 25 mm × 50 mm smooth control films, because it provided an adequate number of CFUs (around 50 well defined CFUs per film using the agar contact method described in the *Sampling and colony counting* section) to have statistical difference between surfaces with or without antibacterial activity. As described above, one of the modifications made was the addition of glycerol 5% to the bacterial suspension to reduce the surface tension of the PBS on the film, which allowed a more homogeneous distribution of the bacterial suspension on the surface. A 25 mm × 50 mm control film and patterned film specimens were placed together in a sterile 90 mm Petri dish (Fig. 3b). A sterile velveteen cloth was placed on a replica plating tool (Bel-Art Products, Wayne, NJ) (Fig. 3c) and immersed into 10 mL of the bacterial suspension by direct contact during 1 min (Fig. 3d). The excess of bacterial suspension was eliminated by placing the inoculated cloth for 10 s onto another dry sterile velveteen cloth (Fig. 3e). The inoculated velveteen cloth was pressed for 10 s contact time against the surface of the films (Fig. 3f). The surfaces were allowed to dry under ambient conditions (± 24 °C and humidity of ± 30%) for 0 min (transference) or 90 min (persistence) (Fig. 3g). Films were sampled (Fig. 3h) and colonies counted as described in the *Sampling and colony counting* section (Fig. 3i).

**Figure 4:**
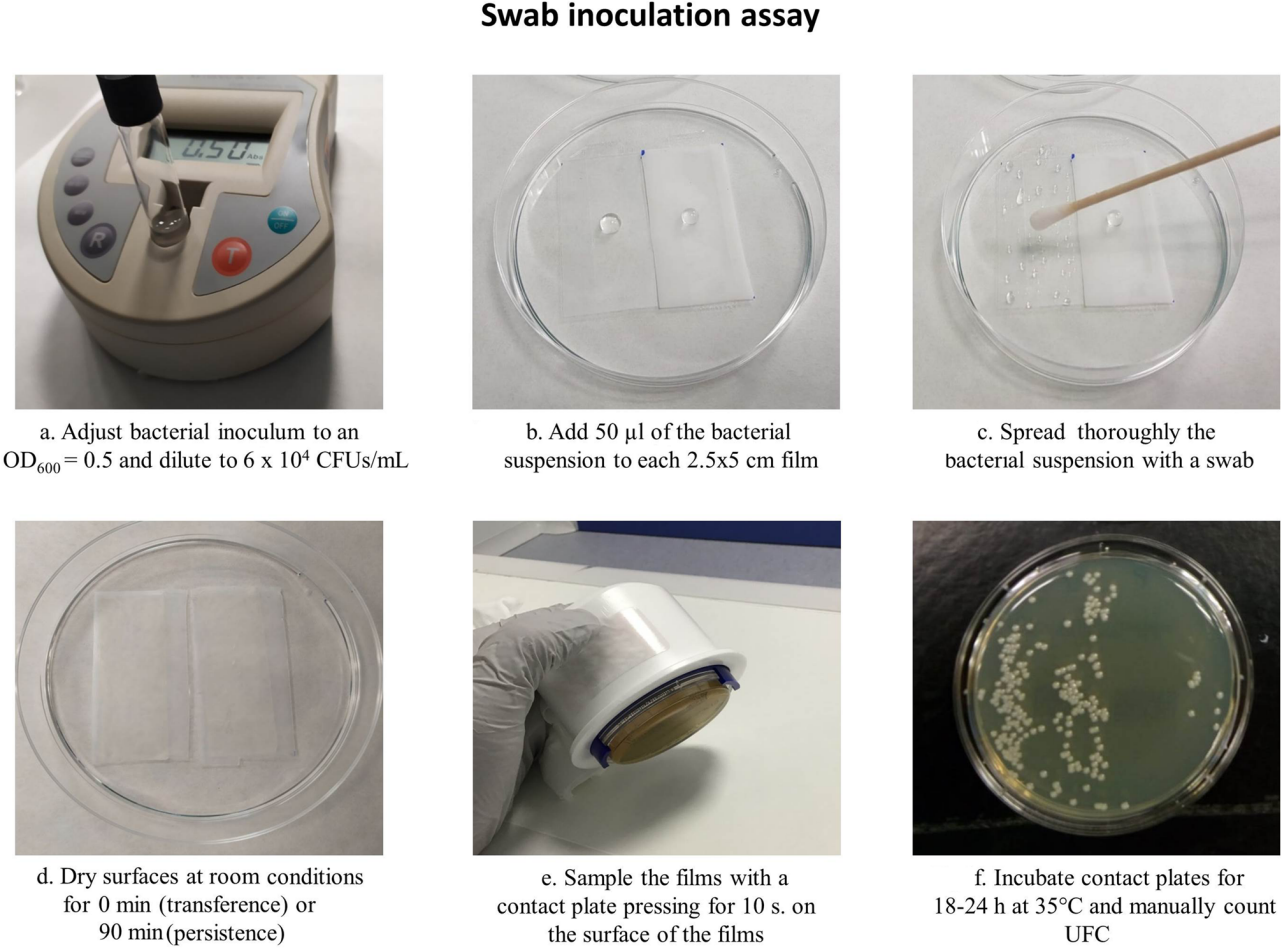
Schematic representation of the swab assay.

### Sampling and colony counting

To quantify the bacteria on the film surface, the agar contact method was used^15^ to directly transfer the bacteria of the surface of the films to the culture agar plates (COUNT-TACT plates, bioMèrieux, France). Contact plates were pressed for 10 s onto the surfaces of the films with the help of a COUNT-TACT applicator (bioMèrieux, France) that applies a uniform pressure of 0.5 kg on the surface of the contact agar plate. After inoculation, plates were incubated for 18–24 h at 35 °C. Colony counting was performed manually. The complete removal of bacteria form film surfaces was confirmed by optical microscopy after simple methylene blue staining and by thoroughly rubbing the surfaces of films with a swab and culturing them, without observing any bacterial growth.

### Data reporting and statistical analysis

For each assay, three samples of each plastic film with micro- and nano-structures and three smooth control samples were analysed. Each assay was performed twice. Mean colony counts and standard deviation (SD) were calculated for each film and controls. Results were expressed as logarithm of CFUs/cm^2^. A single student’s *t*-test was used to compare the bacterial counts between the patterned and the smooth surfaces. A *p*-value lower than 0.05 was considered statistically significant. Statistical analyses were performed using Prism GraphPad version 8 software (GraphPad Software, Inc., La Jolla, CA, USA).

## Results

**Figure 5:**
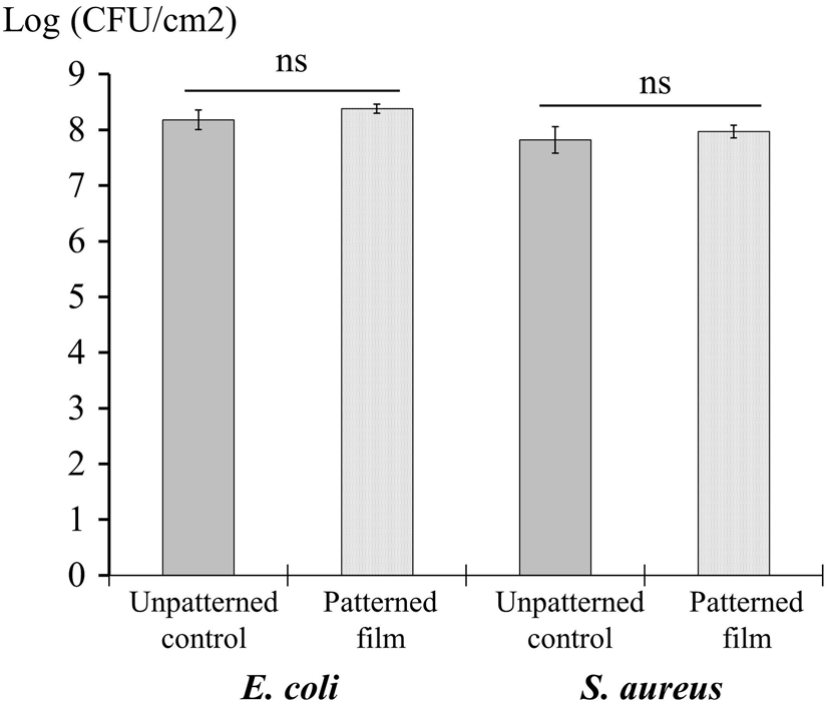
ISO 22196 standard. Antibacterial activity of a patterned film on *E. coli* and *S. aureus* according to the ISO 22196 standard. Bars represent average bacterial counts expressed as Log(CFU/cm^2^). *ns* no significant.

The antibacterial activity of the micro- and nano-patterned film against *E. coli* and *S. aureus* was assessed by the four described methodologies. In the ISO 22196 assay (Fig. 5), the inoculum had to be spread over the surface of the film with the help of a piece of Stomacher bag. During the performance of the assay, it was observed that due to the hydrophobicity of the film it was difficult to spread the inoculum homogenously over the surface leaving some parts of the film not in contact with the bacterial inoculum. As shown on Fig. 4, no reduction in the bacterial count was observed on the patterned film compared to the smooth control film after 24 h of incubation in any of the assays.

**Figure 6:**
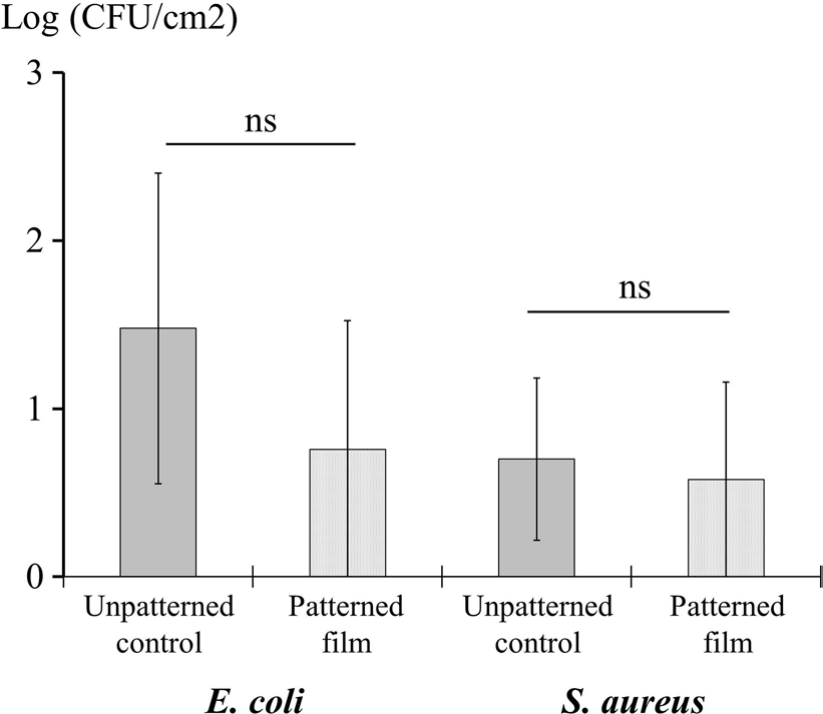
Immersion assay. Anti-attachment activity of a PP patterned film on *E. coli* and *S. aureus* studied by the immersion method. Bars represent average bacterial counts expressed as log (CFU/cm^2^). *ns* no significant.

**Figure 7:**
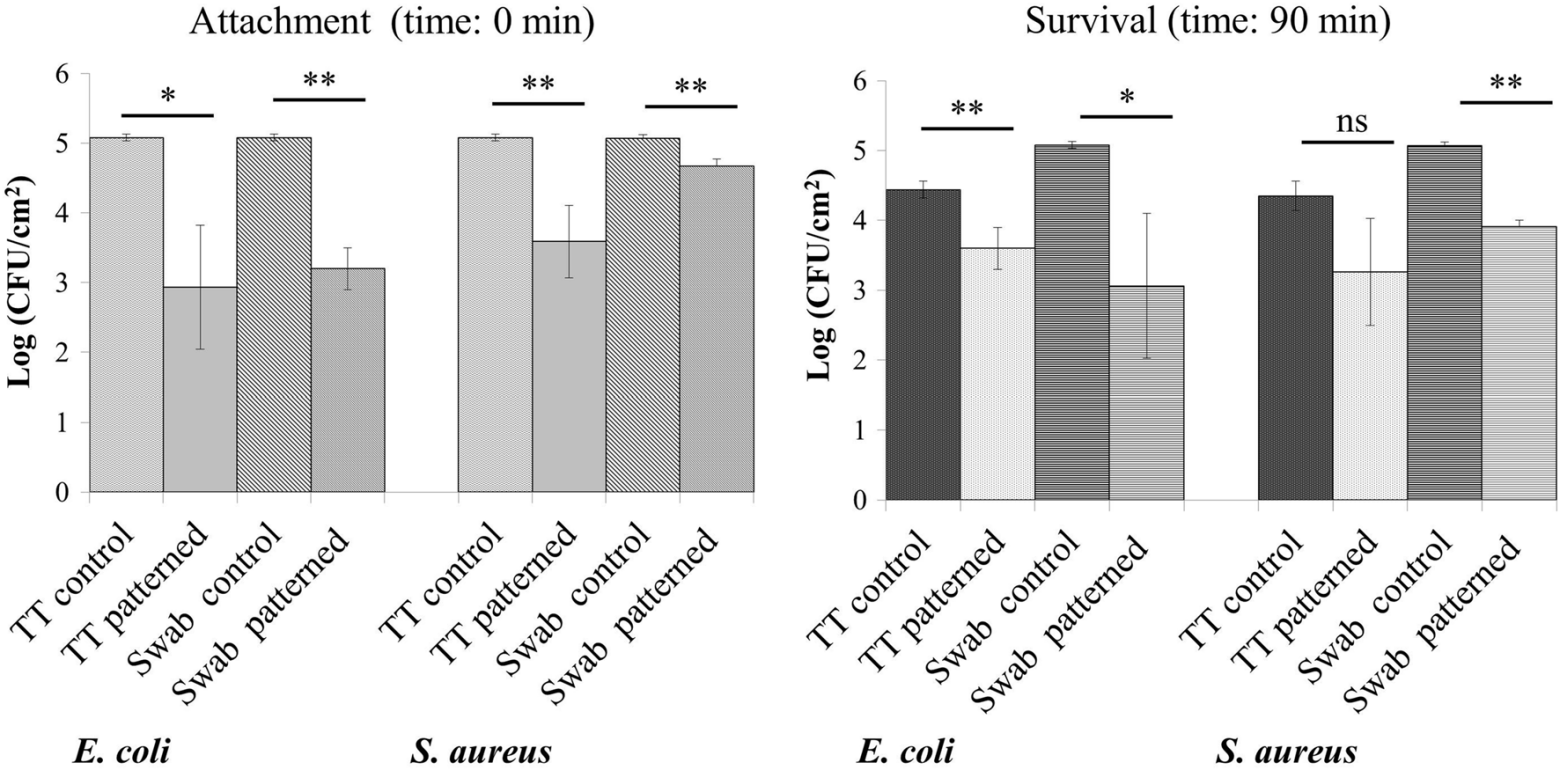
Touch-transfer (TT) and Swab assays. Attachment and survival of *E. coli* and *S. aureus* on a PP patterned film after 0 and 90 min of incubation, respectively, according to the Touch-transfer and Swab assays. Bars represent average bacterial counts expressed as log (CFU/cm^2^). *ns* no significant. *p < 0.05. **p < 0.01.

The bacterial attachment to the film was also tested by the immersion assay (Fig. 6). The bacterial counts in the patterned film were not statistically lower than the counts on the control film (p > 0.05 for all assays). However, close to zero attachment of bacteria was observed in the control film with this method (Fig. 6: 23 CFU/cm^2^) as compared to the touch-transfer and swab-transfer inoculation assays (Fig. 7: 10^5^ CFU/cm^2^).

The Touch-transfer and the Swab inoculation assays (Fig. 7) were performed to test the attachment of bacteria (after 0 min of incubation) to the surface and the antibacterial activity (after 90 min of incubation) of the film against *S. aureus* and *E. coli*.

The bacteria inoculum was prepared with and without glycerol 5% in order to spread the inoculum more homogenously over the film. The addition of glycerol 5% showed a better spread of the inoculum over the surface without altering the growth of the bacteria as observed in the control film (data not shown). The bacterial count on control films was very uniform (4.5–5 CFU/cm^2^, standard deviation < 0.15 in all cases).

As depicted on Fig. 7, at time-point 0, on the patterned film, a lower bacterial attachment for *E. coli* and *S. aureus* was observed by both methods (p < 0.05). The antibacterial activity of the film was also studied after 90 min of contact between the bacteria and the surface of the film, either the inoculum was transferred with the help of a velveteen cloth or with a swab. A statistical reduction in bacterial counts was observed in both bacterial species with both methods except for *S. aureus* in the touch-transfer assay, in which the reduction did not reach the statistical significance (p = 0.052; Fig. 7).

A comparison of the conditions and advantages and disadvantages of the four methods used in this study is showed in Table 1.

**Table 1:** Comparison of the conditions and advantages/disadvantages of the four methods studied to test the antimicrobial activity of highly hydrophobic surfaces.

Step	Conditions	Method			
		ISO 22196	Immersion assay	Swab assay	Touch-transfer assay
Incubation	Temperature Humidity	35 °C > 90% of humidity	Room temperature (24 °C) > 90% of humidity	Room temperature (24 °C) 31% humidity	
	Dis/Adv	Not real-life conditions	Not real-life conditions	Better mimic real-life conditions	
Exposition of bacterial inoculum	Time	24 h	1 h	0 and 90 min	
	Dis/Adv	Test the antibacterial activity of the film	Tests the anti-attachment activity of the film	Analyze both the anti-attachment and antibacterial activity of the film	
Bacterial collection after incubation	Method1	Collected by washing with SCDLP broth, serial dilutions, culture in TSA plates	Contact plates	Contact plates	
	Dis/Adv	Time-consuming; higher risk of contamination	Easier way to collect bacteria from the surface of the film One of the classical methods for direct sample collection of healthcare environment surfaces		

*Dis/Adv* disadvantages/advantages, *SCDLP* Soybean Casein Lecithin Polysorbate broth, *TSA* Trypcase Soy Agar supplemented with 5% sheep blood.

## Discussion

Frequently-touched environmental surfaces play an important role as reservoirs and sources of microorganism transmission^10^. Microbial pathogens are able to survive on the surfaces for long time, and could be transferred and cause HAI in patients admitted to hospitals^12^. There are different current strategies to prevent the contamination of surfaces and therefore, reduce the possibility of patients’ infection. Manual cleaning with antibacterial products is the disinfection method most commonly used in healthcare settings. Automated cleaning using UV light is another method that can be employed to provide not only surface but also environmental disinfection in critical areas of the hospitals^6^. More recently, the use of antimicrobial surfaces to permanently prevent their role as reservoir for potential pathogens has emerged as a potential solution^7,8^. Among them, antiadhesive surfaces, contact-active surfaces, biocide-releasing surfaces or modified topographies have been developed^8^. It has been demonstrated that the micro-patterned surface of films reduces bacterial contamination^7^. To evaluate the antibacterial activity of patterned films different tests can be used and depending on the methodology used, the antibacterial activity results could vary^7,16–18^.

One of the most commonly applied tests for the measurement of antibacterial activity on surfaces is the ISO 22196 standard, which evaluates the antibacterial activity of plastic surfaces after the incubation of a known bacterial inoculum for 24 h^11^. However, the usefulness of the ISO 22196 assay for the evaluation of new antibacterial surfaces is unclear as it showed discrepancies with other methods mainly due to the unrealistic way of transferring bacteria to the plastic surfaces, the high inoculum used and the amount of culture medium left in the liquid layer for 24 h for the diffusion of chemical antibacterial products present in plastics^13,18^. In a study carried out in 8 different research facilities, differences between laboratories were found using the ISO 22196 standard method, mainly for materials with intermediate antibacterial activity^17^. Incubation time, initial bacterial concentration, bacterial phase of growth and nutrient concentration were critical factors that influenced the results of antibacterial testing. It has been demonstrated that the incubation conditions used in the ISO 22196 can influence the antibacterial activity of some materials^12,19^. Differences in the activity of silver-ion containing materials were observed when they were tested using the ISO 22196 conditions (high activity at > 90% relative humidity) instead of more realistic conditions of 22% relative humidity (nearly no activity)^19^. However, humidity was not critical for the activity of copper and copper alloys.

In our study, no antibacterial activity of the film was shown by the ISO 22196 method. The conditions used in the ISO 22196 (high inoculum, bacterial culture media and incubation temperature of 35 °C, and relative humidity of 90%) were far away from the conditions observed in the real setting. By using the more realistic touch-transfer and swab inoculation assays the antibacterial activity of patterned film could be demonstrated. The activity of the film was more related to the anti-attachment activity, probably due to its high hydrophobicity, than to a direct killing of the bacteria. The ISO 22196 test is adequate for testing films releasing antibacterial active compounds but not for films whose antibacterial activity is based in their surface structure.

The touch-transfer and the swab inoculation assays, both studying transference and persistence events, were highly reproducible supported by the low standard deviation observed in the bacterial counts of the control film. The swab assay was designed in our laboratory taking the protocol of Mann et al.^7^ as a reference but trying to better standardize the inoculum applied to the film by controlling the amount of bacteria placed on it. Another modification was the addition of glycerol 5% to the inoculum to obtain a better distribution of the bacterial suspension all over the surface of the highly hydrophobic film. Glycerol was selected because it is commonly used as preservative in bacterial freezing media and experimentally it greatly improved the distribution of the inoculum that otherwise, using only PBS, would have been impossible to achieve. Glycerol was innocuous for the bacteria and reduced the surface tension of the bacterial suspension thus achieving a homogeneous distribution of the bacteria on the film. After inoculating the film surfaces, bacteria were left for 90 min at room temperature (24 °C) and humidity (31%) before taking samples with the contact plates better mimicking the conditions found in a hospital ward. In fact, the experiment was performed in the laboratory of a hospital which better represented the environment of the patients’ rooms. Finally, to test the attachment capacity of highly hydrophobic surfaces, the immersion assay revealed not to be as suitable as both the touch-transfer and the swab inoculation methods used since the attachment of bacteria was much lower in the immersion assay than in inoculation methods: 10^1^ in the immersion and 10^4^–10^5^ in the swab inoculation touch-transfer methods, respectively. Nevertheless, it is an easy and practical method to study the bacterial attachment although it does not reflect commonly ways of bacterial contamination of surfaces^7^.

One limitation of our study was that the possible interference of the glycerol added to the bacterial inoculum on bacterial attachment and on the antibacterial activity of patterned films was not determined. Nevertheless, no differences in bacterial attachment were observed between glycerol at 5% and 40%. Also, the same inoculum containing PBS and glycerol was used to test the control (smooth) and patterned surfaces, what should have balanced the possible effects of glycerol on attachment. So, although we could not totally rule out some influence of glycerol on these methods, it seems unlikely. Another limitation was that bacterial attachment patterns and removal from surfaces was not assessed by high-resolution techniques as SEM. The agar contact method has shown to left no bacteria on similar surfaces after SEM analysis^7^.

Comparing all approaches tested, we concluded that the touch-transfer and swab inoculation methods were the most reproducible and the best at mimicking the various aspects of a real-world surface bacterial contamination. These procedures evaluated the antibacterial activity of hydrophobic films under real temperature and humidity conditions. The touch-transfer and the swab assays presented here were not pretended to replace the ISO 22196, but proved to be more realistic than that normative for the analysis of the antibacterial activity of the highly hydrophobic films under study. Therefore, considering these and other studies^12,13^, a new ISO method would be desirable as a reference method to study the antibacterial activity of highly hydrophobic patterned plastic surfaces.

## Data Availability

The datasets during and/or analysed during the current study available from the corresponding author on reasonable request.
